# Supplementary material for: Screening for Antimicrobial Resistance and Genes of Exotoxins in Pseudomonas aeruginosa Isolates from Infected Dogs and Cats in Poland
Source: Antibiotics (Basel). 2023 Jul 24;12(7):1226. doi: 10.3390/antibiotics12071226 (PMC10376396; doi:10.3390/antibiotics12071226)
Supplement: Supplementary file 1 [file antibiotics-12-01226-s001.zip › antibiotics-2427457-supplementary.pdf]

Table S1. Statistical analysis of virulence-factor-encoding genes and selected antibiotics relationship in *Pseudomonas aeruginosa* isolates from cats

	plcH		plcN		lasB		Exo T		Exo U		Exo Y		Exo S		Tox A	
	Chi^2 Pearsona/Fisher's exact test	p	Chi^2 Pearsona/Fisher' exact test	p	Chi^2 Pearsona/Fisher' exact test	p	Chi^2 Pearsona/Fisher's exact test	p	Chi^2 Pearsona/Fisher' exact test	p	Chi^2 Pearsona/Fisher' exact test	p	Chi^2 Pearsona/Fisher' exact test	p	Chi^2 Pearsona/Fisher' exact test	p
ENR	1.167886	F 0.41059	0.72488	0.72488	0.1521004	F 0.52173	0.000000	1.0000	F	F **0.05465**	F	F 0.61326	F	F 0.36266	0.0008220	0.97713
MAR	0.9442385	0.62368	F	0.22848	F	F 0.07585	0.000000	1.0000	F	F 0.54817	F	F 0.35661	F	F 0.36266	F	F 0.20152
CIP	1.393184	0.23787	0.1477620	0.70068	0.2926587	0.58852	0.000000	1.0000	9.077888	0.00259	3.526272	0.06040	1.856450	0.17304	2.324055	0.12739
PRA	F	F 0.19777	0.1672481	0.19777	F	F 0.18721	0.000000	1.0000	F	F 0.48043	F	F 0.68109	F	0.08961	2.042461	0.15296
CAZ	0.0400258	0.84143	0.0808334	0.77617	0.0361224	0.84926	0.000000	1.0000	0.0400258	0.84143	3.329833	0.06803	1.273752	0.25906	F	0.30659
AK	0.7080294	0.40010	0.0612630	0.80451	F	F 0.69486	0.000000	1.0000	F	F 0.40516	F	F 0.74936	F	F 0.64339	F	F 0.10190
CN	F	F 0.16992	1.395484	0.49771	1.060075	0.58858	0.000000	1.0000	0.0080401	0.99599	0.8577561	0.65124	1.204870	0.54748	1.145845	0.56388
TOB	0.2786775	0.59757	0.0295567	0.86350	0.1916829	0.66152	0.000000	1.0000	0.2786775	0.59757	0.1505582	0.69800	0.2343595	0.62831	0.0493189	0.82425
TTC	0.2786775	0.59757	0.0295567	0.86350	0.1916829	0.66152	0.000000	1.0000	0.2786775	0.59757	0.1505582	0.69800	0.2343595	0.2343595	0.0493189	0.82425
IMP	0.0035755	0.95232	3.741770	**0.05307**	0.1933460	0.66015	0.000000	1.0000	0.0035755	0.95232	0.6730838	0.41198	0.0550237	0.81454	0.2204844	0.63867
MEM	0.2786775	0.20156	1.631047	0.20156	0.1916829	0.66152	0.000000	1.0000	2.879193	0.08973	0.1505582	0.69800	0.2343595	0.62831	0.0493189	0.82425

F - Fisher' exact test

Table S2. Statistical analysis of virulence-factor-encoding genes and selected antibiotics relationship in *Pseudomonas aeruginosa* isolates from dogs

	plcH		plcN		lasB		Exo T		Exo U		Exo Y		Exo S		Tox A	
	Chi^2 Pearsona/Fisher's exact test	p	Chi^2 Pearsona/Fisher's exact test	p	Chi^2 Pearsona/Fisher's exact test	p	Chi^2 Pearsona/Fisher's exact test	p	Chi^2 Pearsona/Fisher's exact test	p	Chi^2 Pearsona/Fisher's exact test	p	Chi^2 Pearsona/Fisher's exact test	p	Chi^2 Pearsona/Fisher's exact test	p
ENR	F	F 0.39229	1.149626	0.56281	1.218441	0.54377	1,163848	0.55882	0.9487138	0.62229	0.4774660	0.78763	3.831767	0.14721	0.1545833	0.92562
MAR	2.603961	0.27199	1.754065	0.41602	1.374084	0.50306	1.712011	0.42486	5.013006	**0.02516**	1.492501	0.47414	0.5536341	0.75819	3.102259	0.21201
CIP	4.650666	0.09775	6.513403	**0.03852**	1.438495	0.48712	0.1611258	0.92260	2.007633	0.36648	1.218598	0.54373	2.925489	0.23160	1.353757	0.50820
PRA	0.9996161	0.60665	0.0965085	0.95289	0.2674198	0.87484	4.178941	0.12375	0.0822036	0.95973	0.0478693	0.97635	1.176365	0.55534	0.0896585	0.95616
CAZ	3.385887	0.06576	0.0000104	0.99743	0.1895303	0.66331	0.0920050	0.76164	0.2168312	0.64147	1.876794	0.17070	1.529459	0.21619	6.247899	**0.01243**
AK	0.2451281	0.88465	2.569788	0.27668	F	F 0.62019	0.5040618	0.77722	3.658822	**0.05577**	5.674916	**0.05857**	3.348414	0.18746	1.150696	0.56251
CN	5.336661	0.06937	1.871062	0.39238	3.485769	0.17501	3.148950	0.20712	0.1659224	0.92039	13.07918	**0.00145**	0.8599455	0.65053	3,289889	0.19302
TOB	0.2623508	0.60851	0.7358660	0.39099	1.108923	0.29232	0.6260647	0.42880	0.0339416	0.85383	1.223788	0.26862	0.3481817	0.55514	2.976780	0.08447
TTC	0.0001572	0.99000	0.1536531	0.69507	1.516088	0.21821	0.7359653	0.39096	0.5189862	0.47127	1.056282	0.30406	1.179914	0.27737	2.569334	0.10895
IMP	0.0075487	0.93076	0.0318507	0.85836	1.472842	0.22490	0.7149719	0.39780	0.7149719	0.39780	0.0407150	0.84009	0.9232876	0.33661	3.191189	0.07404
MEM	0.0035678	0.95237	2.141396	0.14337	0.2556912	0.61310	,3379220	0.56103	0.7812013	0.37677	0.0862958	0.76894	0.0875506	0.76731	0.7936363	0.37300

F - Fisher' exact test
